# Repetitive Electroencephalography as Biomarker for the Prediction of Survival in Patients with Post-Hypoxic Encephalopathy

**DOI:** 10.3390/jcm11216253

**Published:** 2022-10-23

**Authors:** Laurent M. Willems, Felix Rosenow, Susanne Knake, Isabelle Beuchat, Kai Siebenbrodt, Michael Strüber, Bernhard Schieffer, Konstantinos Karatolios, Adam Strzelczyk

**Affiliations:** 1 Department of Neurology and Epilepsy Center Frankfurt Rhine-Main, Goethe-University Frankfurt am Main, 60323 Frankfurt am Main, Germany; 2 LOEWE Center for Personalized Translational Epilepsy Research (CePTER), Goethe-University Frankfurt am Main, 60323 Frankfurt am Main, Germany; 3 Department of Neurology and Epilepsy Center Hessen, Philipps-University Marburg, 35037 Marburg, Germany; 4 Department of Neurology, Centre Hospitalier Universitaire Vaudois (CHUV), University of Lausanne, 1011 Lausanne, Switzerland; 5 Department of Cardiology, Philipps-University Marburg, 35037 Marburg, Germany

**Keywords:** intensive care, neurocritical care, epilepsy, seizures, resuscitation, cardiac arrest

## Abstract

Predicting survival in patients with post-hypoxic encephalopathy (HE) after cardiopulmonary resuscitation is a challenging aspect of modern neurocritical care. Here, continuous electroencephalography (cEEG) has been established as the gold standard for neurophysiological outcome prediction. Unfortunately, cEEG is not comprehensively available, especially in rural regions and developing countries. The objective of this monocentric study was to investigate the predictive properties of repetitive EEGs (rEEGs) with respect to 12-month survival based on data for 199 adult patients with HE, using log-rank and multivariate Cox regression analysis (MCRA). A total number of 59 patients (29.6%) received more than one EEG during the first 14 days of acute neurocritical care. These patients were analyzed for the presence of and changes in specific EEG patterns that have been shown to be associated with favorable or poor outcomes in HE. Based on MCRA, an initially normal amplitude with secondary low-voltage EEG remained as the only significant predictor for an unfavorable outcome, whereas all other relevant parameters identified by univariate analysis remained non-significant in the model. In conclusion, rEEG during early neurocritical care may help to assess the prognosis of HE patients if cEEG is not available.

## 1. Introduction

With an annual incidence of 66 per 100,000 individuals, cardiac arrests (CAs) are frequent critical emergencies and reasons for hospitalization in neurocritical care units (NCUs). In addition to their high mortality rate of approximately 70%, CAs are often accompanied by severe neurological disability in survivors [1,2,3], and non-traumatic out-of-hospital cardiac arrest has been identified as the leading cause of annual disability-adjusted life years (DALYs) in the US [4,5]. After successful cardiopulmonary resuscitation (CPR), patients are usually treated in interdisciplinary intensive care units or NCUs and undergo further diagnostic evaluation to address prognoses and treat possible complications, such as acute symptomatic seizures or status epilepticus (SE). Moreover, patients may undergo therapeutic temperature management (TTM) for 24 h for neuroprotection [6]. In patients who do not adequately recover after TTM and cessation of sedation, hypoxic encephalopathy (HE) is a frequent diagnosis [7,8]. 

Prognostication of HE is a central but difficult aspect of acute neurocritical care and represents an interdisciplinary challenge for NCU physicians [9]. In addition to structural cerebral imaging, perfusion measurement, evoked potentials, and neuron-specific enolase (NSE) measurement 72 h post-CPR, continuous electroencephalography (cEEG) has been established as a valuable predictive tool [3,10,11,12,13,14]. During cEEG or single EEG recordings, abnormal background activity (BA), a burst-suppression pattern (BSP), a low-voltage (LV) amplitude <20 µV, and absence of reactivity have been shown to be associated with an unfavorable prognosis and high short-term mortality, while epileptiform discharges (EDs) and seizure patterns (SPs) have not been clearly associated with an unfavorable outcome [10,11,12,13,14,15,16,17,18]. 

Unfortunately, the availability of cEEG is usually limited to maximum care providers or large specialized hospitals and is not comprehensively accessible, especially in rural areas and developing countries [19]. In addition, extraordinary medical circumstances, such as the recent SARS-CoV-2 pandemic, can significantly restrict the availability of cEEG at neurocritical care centers [20]. In situations in which cEEG is not available or cEEG capacities are exhausted, repetitive EEGs (rEEGs) during acute care may serve as possible alternatives. To date, the prognostic values of changes between early rEEG recordings during the first 14 days after admission have not been adequately addressed, despite evidence for high discriminative potential [21,22,23,24]. 

The aim of this retrospective analysis was to address the prognostic properties of rEEG in adult HE patients, focusing on general changes between two EEGs in the first 14 days of neurocritical care. In addition, the presence and dynamics of specific EEG patterns that have been revealed to be predictive of short-term survival were analyzed. Moreover, the data set was analyzed with respect to sociodemographic and disease-specific aspects that could have favored the implementation of rEEG in the present study population as well as the impact of rEEG compared to single EEG recordings on 12-month survival.

## 2. Materials and Methods

### 2.1. Study Setting and Design

This retrospective analysis is based on data from a large, retrospective, single-center study on EEG findings in SE and encephalopathies [25,26]. Using the hospital information system (HIS) and the hospital EEG database (ED), we identified all adult patients (≥18 years of age) treated at any intensive care unit (ICU) at the University Hospital Marburg (UKGM), Germany, between January 2011 and June 2015 with confirmed HE after CPR. Cases with uncertain HE diagnosis or those that lacked EEG recordings during acute neurocritical care were excluded from the analysis. For later analysis, EEG findings and sociodemographic and clinical variables were extracted from the HIS and ED and were processed. 

For this retrospective analysis, all patients who received at least two EEGs during the first 14 days after CPR were eligible. The interval of two weeks complies with the usual duration of acute neurocritical care and allows for the development of significant changes between EEGs. In patients with more than two EEG recordings during this period, the first and last scalp EEGs recorded were analyzed. 

To improve the readout and interpretation of this study, Strengthening the Reporting of Observational Studies in Epidemiology (STROBE) and REporting of studies Conducted using Observational Routinely-collected Data (RECORD) guidelines were closely followed [27,28]. The initial study and its secondary analysis were approved by the local ethics committee at UKGM Marburg. Due to the retrospective design of the study and the anonymous storage and evaluation of clinical data, informed consent was waived. 

### 2.2. Patient Care and EEG Recordings

Therapy in the ICU was administered according to current national and international guidelines [29,30]. The decision for or against TTM was made individually at the discretion of the treating physician. All EEG recordings were performed after discontinuation of TTM and after a minimum 24 h sedative-free interval, following the recommendations of the German Society for Clinical Neurophysiology (Deutsche Gesellschaft für Klinische Neurophysiologie und Funktionelle Bildgebung (DGKN); Darmstadt, Germany). EEG reactivity was evaluated as reproducible change in EEG frequency or modulation in response to an adequate auditory or nociceptive stimulus. Each EEG was recorded over at least 30 min using the international 10–20 electrode placement system with 21 EEG sintered Ag/AgCl electrodes and rescored independently by at least two experienced, board-certified neurologists following the American Clinical Neurophysiology Society (ACNS, Milwaukee, WI, USA) guidelines [31,32]. If a consensus on the findings could not be reached, a third reviewer was consulted, and a consensus was reached based on the majority principle. According to ACNS guidelines, normal BA was defined as symmetric posterior dominant alpha, beta, theta, or delta activity with reactivity to eye opening or stimuli [31,32]. Deviations from this definition were considered abnormal BA. The definition of SP followed the ACNS definitions of electrographic and electroclinic seizure activity [31,32]. Due to their rare documentation in the present subcohort with rEEGs of the initial study population, rhythmic and periodic pattern (RPPs) were not further analyzed, e.g., lateralized or generalized rhythmic delta activity, lateralized or generalized periodic discharges, or stimulus-induced rhythmic, periodic, or ictal-appearing discharges [33].

### 2.3. Outcome Measures and Statistical Analysis

The main outcome measure of the analysis was survival respectively mortality at 12 months after CPR, which has been established as a feasible outcome interval in post-CPR patients [34]. The data collected for the study did not allow for the differentiation of the patients’ functional outcomes. Moreover, survival and mortality rates at 365 days post-CPR were calculated. 

For the general trend score of rEEG, the parameters of BSP, LV, normal BA, EDs, SP, and EEG reactivity were analyzed. In case of deterioration of one of the parameters (e.g., first occurrence in the last EEG), the trend was classified as “worse”; in case of improvement in at least one and otherwise unchanged parameters, the trend was classified as “improved”. If there was no change between the first and last EEGs, the findings were classified as “stable”. Four states of change were possible for the parameter changes: (1) EEG parameter present in the first but not in the last EEG, (2) EEG parameter present in the first and last EEG, (3) EEG parameter present in the last but not in the first EEG, or (4) EEG parameter present neither in the first nor the last EEG. Due to differences in the significance of individual parameters with respect to malignancy [35], the color coding in Figure 3 was adjusted accordingly (e.g., BSP in both EEGs is a pathological finding, whereas BA reactivity in both EEGs is a benign finding). 

Univariate survival analysis was performed using the log-rank test, and a multivariate Cox regression analysis (MCRA) was used for multivariate analysis. In addition, Pearson’s chi-square test and bivariate Spearman correlation were used to compare nominal or continuous or interval-scaled variables, where appropriate. A *p*-value < 0.05 was regarded as significant. Statistical comparison was performed using SPSS, v.28 (IBM Corp., Armonk, NY, USA) and Prism 7 (GraphPad Software, San Diego, CA, USA), and figures were created using Pixelmator Pro (Pixelmator Team Ltd., Vilnius, Lithuania). A list of abbreviations is available as a supplemental file. 

### 2.4. Analyzed Sociodemographic and Disease-Related Variables and Scale Levels

For statistical analysis, different sociodemographic and disease-related variables with different scale levels were analyzed. Variables with nominal levels were sex, TTM, CPR setting, CPR etiology, and presence of distinct EEG pattern. Variables with ordinal levels used within the analysis were the modified Rankin Scale (mRS), the Charlson Comorbidity Index (CCI), and different states of brainstem reflexes (BSR). Ratio-scaled variables within the analysis were time of survival, age, CRP duration, number of acquired EEGs, time to first and last EEG, as well as length of hospital stay (LOS).

## 3. Results

### 3.1. Univariate Analysis of Reasons for More than One EEG Recording during Acute Neurocritical Care

**Figure 1:**
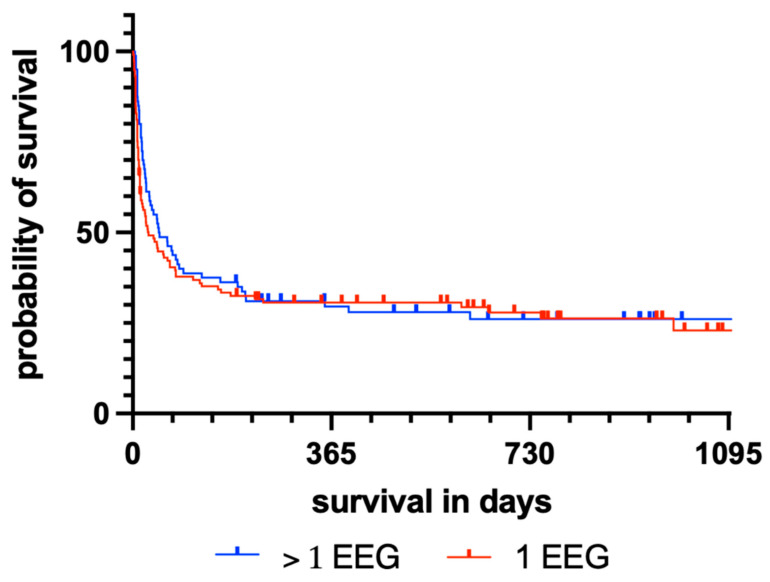
Kaplan–Meier diagram illustrating the survival of HE patients after CPR, taking into consideration the number of derived EEGs.

The overall survival in days did not vary among patients who received one or more EEGs during the first 14 days of acute neurocritical care (*p* = 0.379), with a mean survival of 71.7 days (±18.3 days, median: 16 days, range: 3–993 days) and 65.3 days, respectively (±14.6 days, median: 23 days, range: 3–619 days). Figure 1 shows a Kaplan–Meier survival diagram comparing both subgroups of HE patients. The mortality rate at 12 months after CPR was 74.1% (n = 83) among patients who received only one EEG and 74.7% (n = 56) in patients who received rEEGs. Regarding relevant clinical, disease-specific, and sociodemographic factors, there was no association between the factors and the performance of more than one EEG in the present cohort (Table 1). Regarding known benign and malignant EEG patterns on the first EEG in HE, patients with preserved BA (*p* = 0.017), reactivity (*p* = 0.007), and BSP (*p* < 0.001) received significantly more rEEGs, whereas those with LV received significantly fewer rEEGs. The presence of EDs or SP on the first recorded EEG was not associated with increased performance of multiple EEG recordings (*p* = 0.523 and *p* = 0.517, respectively). None of the patients with continuous EEG suppression after discontinuation of sedation and TTM received more than one EEG. For details, please refer to Table 1.

### 3.2. Sociodemographic and Disease-Specific Characteristics

Of the initial 199 patients, a total of 59 (29.6%) met the inclusion criteria for this analysis. The mean age of the study population was 64.7 years (±11.7 years, median: 65 years, range: 36–87 years), with 39% female and 61% male patients. A median of two EEGs were recorded per patient (mean: 2.9 ± 1.5 EEGs, range: 1–9 EEGs), of which the first EEG was recorded on median day 4 (mean: 4.3 ± 2.7 days, range: 1–11 days) and the last EEG was derived on median day 10 (mean: 9.5 ± 3.3 days, range: 3–14 days). For further information on sociodemographic and disease-specific characteristics, please refer to Table 2.

### 3.3. Univariate Analysis of the Predictive Properties of Repetitive EEGs during Acute Neurocritical Care

**Figure 2:**
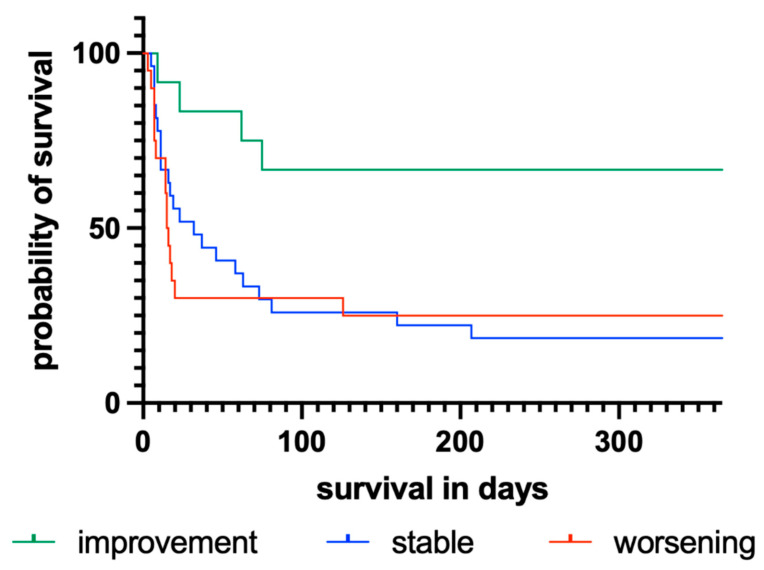
Kaplan–Meier diagram illustrating the influence of an improved, stable, or worsening outcome of the last EEG compared to the first EEG within 14 days after CPR.

**Figure 3:**
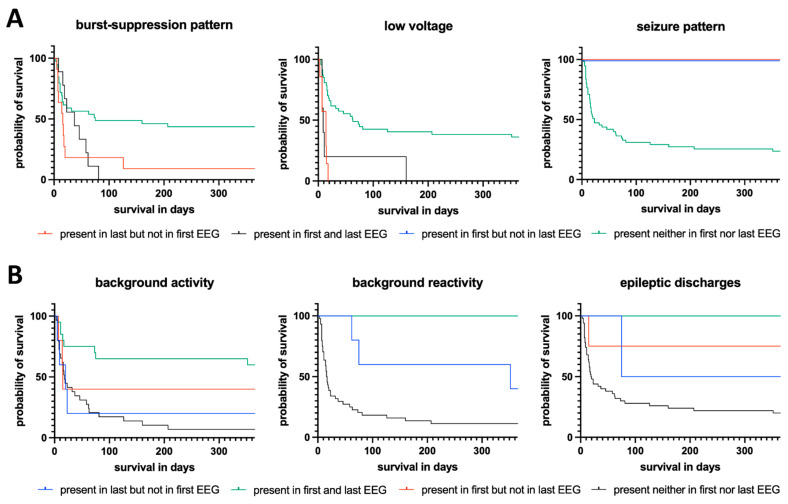
Kaplan–Meier survival diagram illustrating the influence of altered EEG findings between the first and last EEGs within 14 days after CPR in adult patients with HE. The log-rank test showed a significant difference between the subgroups in terms of 12-month survival for all examined EEG features, except for seizure pattern (see Table 3). Due to the different benignity or malignity of the existing EEG pattern, the color of the legend changes between the subfigures (**A**) and (**B**) (see Section 2.3).

By applying a simple scoring system of improvement, worsening, or constant findings between the first and last recorded EEGs, a significant difference regarding 12-month survival was revealed (*p* = 0.036). With a mean survival of 256 days (median: 365 days) compared with 102 (median: 32 days) or 105 days (median: 16 days), patients showing an improved EEG within the first 14 days after CPR had more than twice the median survival of patients with constant or worsening findings, respectively (Table 3). This relationship is presented in the Kaplan–Meier diagram shown in Figure 2. In addition, the log-rank test revealed a significant difference between the subgroups in terms of 12-month survival for BSP (*p* = 0.016), LV (*p* < 0.001), BA (*p* = 0.002), reactivity (*p* < 0.001), and ED (*p* = 0.031), but not for SP (*p* = 0.058). The results of univariate log-rank analysis of the prognostic properties of general changes and specific findings between the first and last EEGs recorded during the first 14 days after CPR and the individual mortality rates are presented in Table 3. The corresponding Kaplan–Meier graphs for overall changes are provided in Figure 2 and for BSP, LV, BA, reactivity, ED, and SP in Figure 3.

### 3.4. Multivariate Analysis of Predictive Properties of Repetitive EEGs during Acute Neurocritical Care

The final MCRA model predicted 12-month survival significantly more effectively than the univariate analysis (*p* < 0.001, chi = 42.887). Out of all the included parameters, only the finding of an LV (amplitude <20 µV) in the last EEG in patients with previously normal EEG amplitude remained significant (*p* = 0.004). Please refer to Table 4 for further details. 

## 4. Discussion 

This retrospective monocenter study analyzed the prognostic properties of rEEG in adult patients with HE after CPR regarding 12-month mortality and survival as an alternative monitoring option in the absence of cEEG capacities. For a reliable evaluation, the focus of the analysis was set on changes between the first and last EEGs, recorded within the first 14 days on NCU. Both general EEG changes as well as the prevalence and improvement or worsening of distinct EEG patterns associated with high 12-month mortality were investigated. In summary, it was demonstrated that rEEG has a certain legitimacy in the prognostication of critically ill patients with HE after CPR. Although in the univariate analysis some distinct EEG patterns and their course were associated with higher mortality after 12 months, only meeting the criterion of low-voltage EEG in the first EEG or during acute critical care remained a significant predictor according to the MCRA. In the following, we will discuss the findings of the univariate and multivariate analyses in detail and frame them in the context of cEEG and acute-care patients with HE after CPR.

Based on the current knowledge of the prognostic properties of distinct EEG findings in HE patients, the present analysis focused on BSP, LV, SP, BA, EEG reactivity, and ED; however, the focus was not solely on the presence but on the dynamics of the changes in these patterns [16,36,37,38,39,40,41,42]. For all patterns except SP, the dynamic between the first and last EEG recorded within 14 days of acute neurocritical care had a significant impact on 12-month survival according to the univariate analysis by log-rank test (Table 3, Figure 3). In line with previous publications, the presence or development of low-voltage EEG, especially, as well as missing reactivity in the first or last EEG were significantly associated with high 12-month mortality [3,16,36,37,38,43], while the presence of a reactive EEG at the first or last EEG was associated with high 12-month survival rates. Notably, none of the patients with reactivity initially or preserved during the course died during the first 12 months after CPR, which is consistent with other publications [16,36,37,38]. With regard to a normal BA, absence in the first and last EEG and occurrence in only one of the two EEGs was associated with higher mortality, which seems comprehensible in the context of other publications highlighting a normal BA as prognostically favorable [16,36,37,38]. The initial or secondary presence of BSP was also a strong predictor for 12-month mortality, even if the prognostic properties of BSP have been controversial [39,40,41,42]. As reported previously, the initial or secondary proof of ED or SP was associated with lower 12-month mortality; however, in the present study population, only the presence of ED reached a level of significance [44,45,46,47]. 

Based on the already mentioned EEG patterns, e.g., BSP, LV, BA, reactivity, ED, and SP, a descriptive EEG score was developed and analyzed for its association with higher 12-month survival. Based on the simple dynamic of EEG findings, the score distinguished patients with stable, improved, or worsened findings, comparing the last with the first EEG recorded during the first 14 days of neurocritical care. Patients with an improvement in EEG over time showed significantly higher 12-month survival (*p* = 0.036) compared to patients with stable or worsening EEG findings (Figure 2, Table 3). In line with previous studies, these results highlight the diagnostic benefit of rEEG in HE patients after CPR [21,48].

To further analyze these results, an MCRA was performed, resulting in a model that was superior in predicting 12-month survival compared to the univariate analysis. Here, LV EEG with initial normal amplitude was the only EEG pattern that remained significant for the prediction of 12-month survival (Table 4). Hence, LV appeared to be the strongest prognostic predictor in the studied patient population. Although all other analyzed parameters remained non-significant in the Cox regression model, patients with persistent or secondary BSP, persistent LV EEG, and initially or persistently abnormal BA showed high mortality within the first 12 months after CPR, with Exp(B) values between 1.4 and 2.8 (Table 4). These findings underline the previously reported malignant character of these EEG findings and their importance as prognostic factors in patients with HE after CPR [16,36,37,38]. Regarding the interpretability of the MCRA, the limited number of patients and the different characteristics within the study cohort must be considered.

In addition to the previously mentioned EEG findings, studies from recent years have shown that the presence of rhythmic or periodic patterns (RPPs) (e.g., rhythmic delta activity, lateralized or generalized periodic discharges, and stimulus-induced rhythmic, or periodic, or ictal discharges) could be associated with an unfavorable prognosis [49]. Although RPPs were observed quite frequently in the initial population (n = 199), they were not sufficiently prevalent in the study population of patients with rEEGs during acute care to be further evaluated [3].

Interestingly, in the present retrospective evaluation, performing more than one EEG compared with only one EEG had no effect on survival over a 3-year period after CPR (Figure 1). Moreover, there was no significant correlation between sociodemographic or disease-related factors (e.g., age or pre-existing medical conditions) and the performance of rEEG (Table 1). Based on the presumption of higher mortality among patients with CA and HE by physicians, the authors expected a higher proportion of individuals who received only one EEG among older or premorbid patients, which has been reported to cause premature discontinuation of life-sustaining therapies [50,51]. However, the non-significant findings regarding sociodemographic and disease-specific aspects for the cohort indicate that there was no major selection bias.

In line with the few previous studies on this topic, the results of the present analysis suggest that rEEG seems feasible as a neurophysiological biomarker and NCU monitoring option for 12-month survival in adult HE patients [21,48]. Considering the still-limited availability of cEEG in rural regions and developing countries, rEEG is a viable alternative to the gold standard of neurophysiological monitoring in neurocritical care [19]. A recent international survey showed that only a few ICUs have 24/7 availability of EEG technicians and specialized physicians. In most participating centers, adequate coverage with specialized staff was only available on weekdays during regular working hours from 9 AM to 5 PM, which makes the reasonable implementation of cEEG nearly impossible [52]. Additionally, from a financial point of view, rEEG seems to have an advantage over cEEG, since the latter has significantly higher acquisition, operating, and personnel costs not necessarily reflected in the reimbursement of hospital services [53]. The recent SARS-CoV-2 pandemic has also shown how quickly neurocritical care resources at maximum care hospitals, which are already limited, can be depleted or diverted to maintain other systemically important intensive care facilities [20,54,55]. Nevertheless, cEEG should be the gold standard for acute neurocritical care in patients with HE after CPR, as it was associated with increased detection of interictal features and seizures compared to routine EEG recordings [56,57]. Moreover, cEEG was associated with more frequent adaption of anti-seizure medication in critically ill patients, which indicates its high potential in therapy finding and control [56]. Furthermore, cEEG has been revealed to be associated with favorable hospitalization outcomes for critically ill patients in general in terms of in-hospital mortality, treatment costs, and length of stay [56,58].

As with any monocentric retrospective study, this evaluation is subject to certain limitations that may have influenced the results. Out of the initial study population of 199 patients, only 59 patients had more than one EEG recorded within the first 14 days of acute care. The present results can therefore only have an orienting character and need further verification. Despite the treatment of patients according to national and international standards, regional preferences or variations may have influenced acute neurocritical care. In addition, the free decision of the treatment team regarding TTM or the performance of one or more EEGs during acute care may have had an influence on the results. In addition, the self-fulfilling presumption of high mortality among patients with CA and HE by physicians could represent a potential bias. In this study, the data set did not allow for the assessment of functional outcomes, which could potentially have biased the results through survival with low functional status. By using standardized ICU care concepts, performing blinded and cross-verified EEG analyses, and by closely following RECORD [59] and STROBE [60] guidelines, we strived to reduce biases to an acceptable minimum. 

## 5. Conclusions

In conclusion, rEEG recordings during early neurocritical care can help to distinguish between HE patients with benign or malignant prognosis regarding 12-month mortality. If cEEG is not available during acute neurocritical care, rEEG could be a reliable alternative for prognostication after CPR. However, larger studies are needed to confirm and further analyze these findings. 

## Figures and Tables

**Table 1:** Univariate analysis of factors leading to multiple EEG recordings in HE patients (n = 199).

	Rho ^a^			*p*-Value ^a^
Time of survival, days	−0.022			0.060
Age, years	−0.047			0.371
CPR duration, minutes	−0.140			0.214
Modified Rankin Scale (mRS)	0.091			0.072
Charlson Comorbidity Index (CCI)	−0.043			0.397
		1 EEG % (n)	>1 EEG % (n)	*p*-Value ^b^
Intact brainstem reflexes (BSR)	Yes	44.0 (11)	56.0 (14)	0.085
	No	62.1 (108)	37.9 (66)	
Sex	Male	63.2 (84)	36.8 (49)	0.170
	Female	53.0 (35)	47.0 (31)	
Therapeutic temperature management (TTM)	Yes	62.5 (70)	37.5 (42)	0.384
	No	56.3 (45)	43.8 (35)	
CPR setting in hospital	Yes	64.4 (47)	35.6 (26)	0.560
	No	60.2 (71)	39.8 (47)	
EEG suppression (<2 µV) during first EEG	Yes	100.0 (4)	0.0 (0)	0.980
	No	58.9 (115)	41.1 (80)	
Preserved normal background activity during first EEG	Yes	51.0 (48)	49.0 (46)	0.017
	No	67.6 (71)	32.4 (34)	
Low voltage during first EEG	Yes	74.4 (29)	25.6 (10)	0.039
	No	56.3 (90)	43.7 (70)	
Burst-suppression pattern during first EEG	Yes	25.9 (7)	74.1 (20)	<0.001
	No	65.1 (112)	34.9 (60)	
EEG reactivity during first EEG	Yes	70.4 (50)	29.6 (21)	0.007
	No	50.4 (59)	49.6 (58)	
Epileptiform discharges during first EEG	Yes	71.4 (5)	28.6 (2)	0.523
	No	59.4 (114)	40.6 (78)	
Seizure pattern during first EEG	Yes	60.0 (6)	40.0 (4)	0.517
	No	60.3 (114)	39.7(75)	

EEG = electroencephalography; CPR = cardiopulmonary resuscitation; HE = post-hypoxic encephalopathy. ^a^ Calculated using bivariate Spearman correlation. ^b^ Calculated using Pearson’s chi-square test.

**Table 2:** Sociodemographic, length of stay, CPR, and outcome parameters for the study population (n = 59).

Sociodemographic Parameters		
Age, years	Mean ± SD	64.7 ± 11.7
	Median	65.0
	Range	36–87
Sex, % (n)	Female	39.0 (23)
	Male	61.0 (36)
Modified Rankin Scale (mRS)	Mean ± SD	1.1 ± 0.7
	Median	1.0
	Range	0–2
Charlson Comorbidity Index (CCI)	Mean ± SD	4.5 ± 2.5
	Median	4.0
	Range	0–10
CPR parameters		
CPR etiology	Primary cardiac	76.3 (45)
	Primary respiratory	15.3 (9)
	Other	8.4 (5)
CPR duration, minutes	Mean ± SD	23.8 ± 16.7
	Median	20.0
	Range	2.0–90.0
CPR setting	In hospital	27.1 (16)
	Out of hospital	64.4 (38)
	n.a.	8.5 (5)
Therapeutic temperature management (TTM)	Yes	37.3 (22)
	No	62.7 (37)
EEG parameters		
Number of EEGs	Mean ± SD	2.9 ± 1.5
	Median	2.0
	Range	1.0–9.0
Time to first EEG, days	Mean ± SD	4.3 ± 2.7
	Median	4.0
	Range	1.0–11.0
Time to last EEG, days	Mean ± SD	9.5 ± 3.3
	Median	10.0
	Range	3.0–14.0
Hospitalization		
Length of stay, days	Mean ± SD	19.1 ± 14.6
	Median	17.0
	Range	3.0–91.0
Mortality		
Mortality % (n)	In hospital	49.2 (29)
	30 days after discharge	54.2 (32)
	365 days post-CPR	71.2 (42)

CPR = cardiopulmonary resuscitation; SD = standard deviation. EEG = electroencephalogram; n.a. = not available.

**Table 3:** Univariate analysis of predictive properties of rEEG parameters in patients with HE after CPR (n = 59).

Changes between First and Last EEGs	Patients	Mortality	Survival in Days				
	% (n)	Rate, % (n)	Mean	± SD	Med	95% CI	*p*-Value ^a^
General trend							
Improvement	20.3 (12)	38.5 (5)	256.3	43.9	-	-	0.036
Stable finding	45.8 (27)	81.5 (22)	101.2	25.8	32.0	1.5–62.5	
Worsening	33.9 (20)	75.0 (15)	105.9	33.9	15.0	12.1–17.9	
Burst-suppression pattern							
Not in first but in last EEG	18.6 (11)	90.9 (10)	54.8	31.2	16.0	6.3–25.7	0.016
In first and last EEG	15.3 (9)	100.0 (9)	38.9	8.2	37.0	0.0–77.9	
In first but not in last EEG	0.0 (0)	-	-	-	-	-	
Neither in first nor in last EEG	66.1 (39)	59.0 (23)	178.8	20.4	32.0	0.0–73.9	
Low-voltage EEG							
Not in first but in last EEG	11.9 (7)	100.0 (7)	11.1	2.1	14.0	5.8–22.2	<0.001
In first and last EEG	8.5 (5)	100.0 (5)	38.8	30.3	9.0	4.7–13.3	
In first but not in last EEG	0.0 (0)	-	-	-	-	-	
Neither in first nor in last EEG	79.7 (47)	63.8 (30)	162.9	23.7	63.0	24.0–102.0	
Normal EEG background activity							
Not in first but in last EEG	8.5 (5)	80.0 (4)	84.4	62.8	20.0	0.0–43.6	0.002
In first and last EEG	33.8 (20)	40.0 (8)	247.0	35.9	15.0	-	
In first but not in last EEG	8.5 (5)	60.0 (3)	153.2	77.5	18.0	12.9–17.1	
Neither in first nor in last EEG	49.2 (29)	93.1 (27)	62.0	17.7	32.0	12.7–23.3	
EEG reactivity							
Not in first but in last EEG	8.5 (5)	60.0 (3)	243.8	64.1	352.0	0.0–946.7	<0.001
In first and last EEG	16.9 (10)	0.0 (0)	365	-	365	-	
In first but not in last EEG	0.0 (0)	-	-	-	-	-	
Neither in first nor in last EEG	74.6 (44)	88.6 (39)	69.5	17.1	16.0	12.3–19.7	
Epileptic discharges							
Not in first but in last EEG	3.4 (2)	50.0 (1)	220.0	102.5	75.0	-	0.031
In first and last EEG	5.1 (3)	0.0 (0)	365	-	365	-	
In first but not in last EEG	6.8 (4)	25.0 (1)	277.5	75.8	365	-	
Neither in first nor in last EEG	8.5 (50)	80.0 (40)	122.0	20.2	23.0	0.0–46.2	
EEG seizure pattern							
Not in first but in last EEG	3.4 (2)	0.0 (0)	365.0	205.1	-	-	0.058
In first and last EEG	0.0 (0)	-	-	-	-	-	
In first but not in last EEG	3.4 (2)	0.0 (0)	365.0	-	365.0	-	
Neither in first nor in last EEG	93.2 (55)	76.4 (42)	117.6	20.1	23.0	2.3–43.7	

EEG = electroencephalography; CPR = cardiopulmonary resuscitation; HE = post-hypoxic encephalopathy; SD = standard deviation; CI = confidence interval. ^a^ Calculated using a log-rank test.

**Table 4:** Multivariate Cox regression analysis of EEG parameters in patients with HE after CPR.

	B ^a^	Exp(B) ^a^	95% CI of Exp(B) ^a^	*p*-Value ^a^
Burst suppression				
Not in first but in last EEG	0.357	1.4	0.5–4.1	0.509
In first and last EEG	0.674	2.0	0.7–5.8	0.223
Flat EEG <20 µV				
Not in first but in last EEG	1.7	5.2	1.7–15.8	0.004
In first and last EEG	1.0	2.8	0.8–9.7	0.094
Normal EEG background activity				
Not in first but in 2nd EEG	0.862	2.4	0.6–8.9	0.201
In first and last EEG	0.532	1.7	0.6–5.2	0.351
In first but not in last EEG	−0.293	0.746	0.2–3.0	0.683
EEG reactivity				
Not in first but in last EEG	−1.1	0.3	0.1–1.2	0.099
In first and last EEG	−12.9	0.0	0.0–0.0	0.950
Epileptiform discharges				
Not in first but in last EEG	−1.4	0.2	0.0–2.3	0.209
In first and last EEG	−0.4	0.6	0.0–0.0	0.999
In first but not in last EEG	−1.4	0.3	0.0–2.1	0.202
Seizure pattern				
Not in first but in last EEG	−0.432	0.6	0.0–0.0	0.999
In first but not in last EEG	0.0	1.0	0.0–0.0	1.000

^a^ Calculated using a multivariate Cox regression model (*p*-value of the model < 0.001, chi = 42.887). CPR = cardiopulmonary resuscitation; EEG = electroencephalography; HE = hypoxic encephalopathy; B = regression coefficient; Exp(B) = hazard ratio.

## Data Availability

Data available on request due to restrictions of the German General Data Protection Regulation (Datenschutz-Grundverordnung (DGSVO). The data presented in this study are available on request from the corresponding author.

## Abbreviations

ACNS	American Clinical Neurophysiology Society
BA	Background activity
BSP	Burst-suppression pattern
BSR	Brainstem reflex
CA	Cardiac arrest
CCI	Charlson comorbidity index
cEEG	Continuous electroencephalography
CI	Confidence interval
CPR	Cardiopulmonary Resuscitation
DGKN	German Society for Clinical Neurophysiology (Deutsche Gesellschaft für Klinische Neurophysiologie
ED	Epileptiform dischargers
EEG	Electroencephalography
HE	Hypoxic encephalopathy
HIS	Hospital information system
ICU	Intensive care unit
LV	Low voltage
MCRA	Multivariate Cox regression analysis
Med	Median
mRS	Modified rankin scale
n.a.	Not available
NCU	Neurocritical care units
NSE	Neuron-specific enolase
RECORD	REporting of studies Conducted using Observational Routinely-collected Data
rEEG	Repetitive electroencephalography
RPP	Rhythmic and periodic pattern
SARS-CoV-2	Severe acute respiratory syndrome coronavirus type 2
SD	Standard deviation
SE	Status epilepticus
SP	Seizure pattern
STROBE	Strengthening the Reporting of Observational Studies in Epidemiology
TTM	Therapeutic temperature management
UKGM	University Hospital Marburg
